# Complete plastid genome sequence of *Oberonioides microtatantha* (Schltr.) Szlach. (Orchidaceae), an endemic herb in China

**DOI:** 10.1080/23802359.2020.1851150

**Published:** 2021-03-01

**Authors:** Caiqin Liu, Ning Kang, Xin Liu, Yu Chen, Jun Tao, Yue Zhang, Yanyan Zhang, Yunlong Li, Guangda Tang, Yuling Li

**Affiliations:** a Guangdong Xiangtoushan Natural Reserve, Huizhou, China; b Huizhou Forestry Science Research Institute, Huizhou, China; c College of Forestry and Landscape Architecture, South China Agricultural University, Guangzhou, China

**Keywords:** Oberonioides microtatantha, Orchidaceae, plastid genome, phylogenomics

## Abstract

The species *Oberonioides microtatantha*, belonging to the family Orchidaceae, is a small lithophytic herb endemic in south China with significant conservation values. The complete plastid genome sequence of *O. microtatantha* reported here is 144,989 bp in length, with a large single copy (LSC) region of 83,920 bp, a small single copy (SSC) region of 13,063 bp, and a pair of inverted repeat (IRa and IRb) regions of 24,003 bp each. The plastome consists of 95 genes, including 72 protein-coding genes, 4 ribosomal RNA genes, and 19 transfer RNA genes. The overall GC content is 36.81%. Phylogenetic analysis placed *Oberonioides* closet to the genus *Liparis* in Orchidaceae.

*Oberonioides* Szlach belongs to the tribe Malaxideae, subfamily Epidendroideae, family Orchidaceae (Chase et al. 2015). It comprises of only two species, among which the species *O. microtatantha* (Schltr.) Szlach. is a small lithophytic herb endemic in China (Szlachetko 1995; Chen and Wood 2009). It is mainly distributed in southeastern China and is found usually growing on humid and shaded rocks in forests (Chen and Wood 2009). Due to its limited distribution, human collection, loss of habitat, and habitat fragmentation, this orchid species was considered near threatened (NT) according to the IUCN Red List of higher plants in China (http://www.iplant.cn/rep/protlist/4).

Samples of *O. microtatantha* were collected from Guangdong Xiangtoushan Natural Reserve, Guangdong province (China: N23°16′9″, E114°22′26″). Voucher specimen (LYL 305) was deposited in Herbarium of South China Agricultural University (CANT). Total genomic DNA of *O. microtatantha* was extracted from a mature leaf of an individual by using modified CTAB method (Doyle and Doyle 1987). The DNA extracted was directly sequenced using the Illumina HiSeq 2500 Sequencing System. The average read length was 150 bp and the reads were assembled with the software GetOrganelle (Jin et al. 2018). Then the genome obtained was annotated by using online software Geseq (Tillich et al. 2017). The accession number MT559316 was obtained after submitting the annotated plastid genome of *O. microtatantha* to the GenBank.

The whole plastid genome of *O. microtatantha* was 144,989 bp in length, composed by a large single-copy (LSC) region of 83,920 bp, a small single-copy (SSC) region of 13,063 bp, and a pair of inverted repeat (IRa and IRb) regions of 24,003 bp each. The annotated genome comprised 95 genes, including 72 protein-coding genes, 4 ribosomal RNA genes (rrn23, rrn16, rrn5, rrn4.5), and 19 transfer RNA genes. 17 genes were duplicated in the IR regions, including 5 protein-coding genes (rpl2, rpl23, rps19, rps7, ycf2), 4 ribosomal RNA genes (rrn16, rrn23, rrn4.5, rrn5), and 8 transfer RNA genes (trnH-GUG, trnA-UGC, trnI-CAU, trnI-GAU, trnL-CAA, trnN-GUU, trnR-ACG, trnV-GAC). The overall GC content of *O. microtatantha* plastid genome was 36.81% (LSC, 34.1%; SSC, 29.31%; IRs, 43.59%).

To investigate the phylogenetic location of *O. microtatantha* within the tribe of Malaxideae, a phylogenetic tree was constructed. The chloroplast genome of *O. microtatantha* was aligned with 15 other complete chloroplast genomes of Malaxideae, and *Acampe rigida*, *Tainia dunnii*, and *Calanthe sylvatica* of Orchidaceae family as outgroup, using MAFFT ver. 7 (Katoh and Standley 2013). Phylogenetic analysis was conducted based on maximum likelihood (ML) analyses using RAxML (Stamatakis 2014). Support for the inferred ML tree was inferred by bootstrapping with 1000 replicates and with GTRGAMMA as the nucleotide substitution model. Phylogenetic analysis results suggests that *Oberonioides* is closely related to the genus *Liparis* (Figure 1). The chloroplast genome of *O. microtatantha* will provide useful genetic information for further study on genetic diversity and conservation of Orchidaceae species.

**Figure 1.**
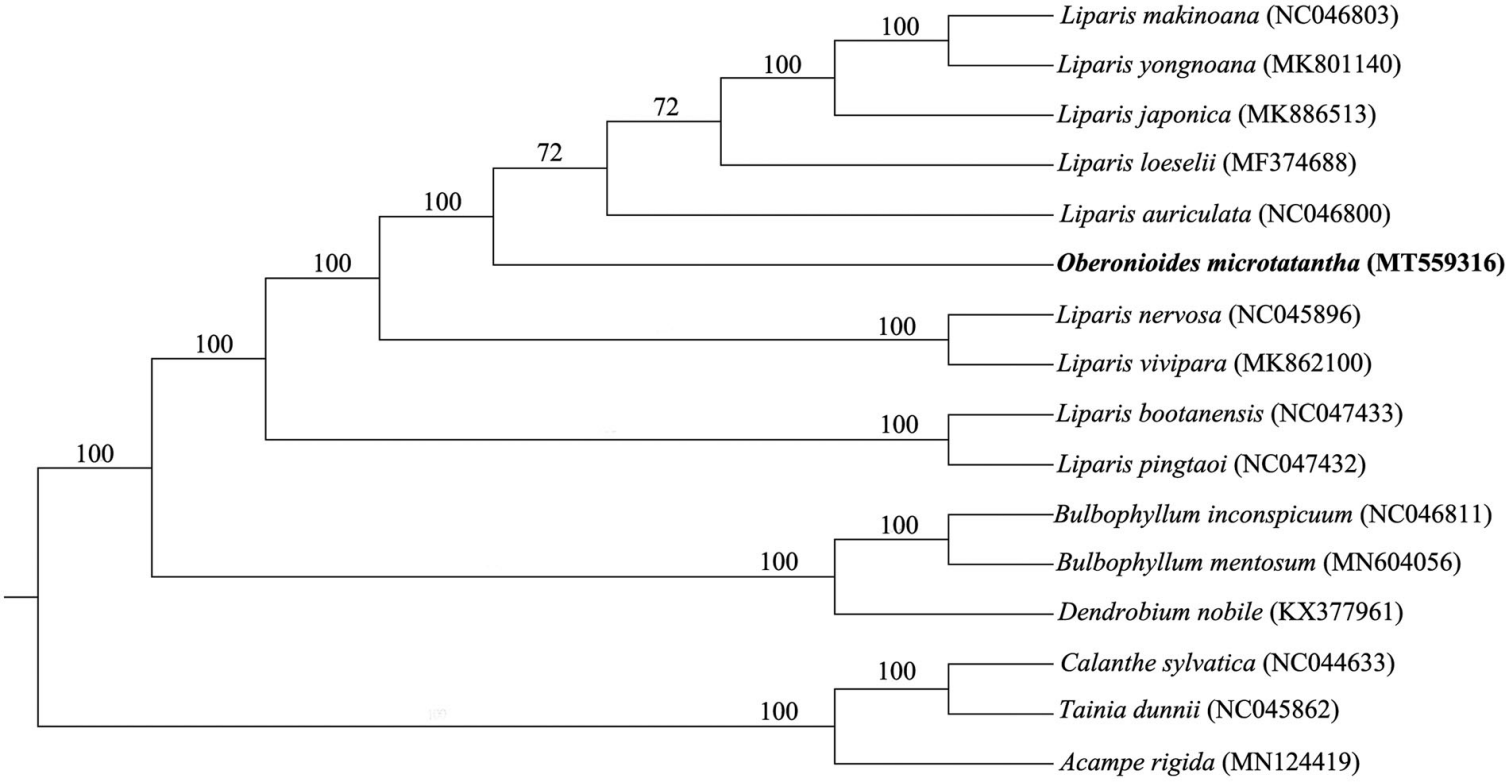
Maximum likelihood tree of tribe Malaxideae based on complete chloroplast genomes, with *Acampe rigida*, *Tainia dunnii*, and *Calanthe sylvatica* as outgroup. Bootstrap support values (based on 1000 replicates) are shown above branches.

## Data Availability

The data that support the findings of this study are openly available in GenBank of NCBI at https://www.ncbi.nlm.nih.gov, reference number MT559316.
